# Hepatitis C Prevalence and Birth Outcomes among Pregnant Women in the United States: A 2010–2020 Population Study

**DOI:** 10.3390/pathogens13040321

**Published:** 2024-04-14

**Authors:** Paul Wasuwanich, Songyos Rajborirug, Robert S. Egerman, Tony S. Wen, Wikrom Karnsakul

**Affiliations:** 1 University of Florida College of Medicine, Gainesville, FL 32610, USA; 2 Department of Epidemiology, Johns Hopkins University Bloomberg School of Public Health, Baltimore, MD 21205, USA; 3 Division of Maternal-Fetal Medicine, Department of Obstetrics & Gynecology, University of Florida College of Medicine, Gainesville, FL 32610, USA; 4 Division of Pediatric Gastroenterology, Hepatology, and Nutrition, Department of Pediatrics, Johns Hopkins University School of Medicine, Baltimore, MD 21205, USA

**Keywords:** epidemiology, liver cirrhosis, seroepidemiologic studies, public health

## Abstract

Background: The rates of hepatitis C virus (HCV) infection have increased in the pregnant population. We aim to describe the age-stratified clinical outcomes and trends for inpatient pregnant women with HCV in the U.S. Methods: We utilized hospitalization data from the 2010–2020 National Inpatient Sample. Pregnancy and HCV were identified according to their ICD-9/ICD-10 codes. Demographic and clinical data including cirrhosis, mortality, preterm birth, and stillbirth were extracted. The age groups were defined as ≤18, 19–25, 26–34, and ≥35 years. Results: We identified 195,852 inpatient pregnant women with HCV, among whom 0.7% were ≤18, 26.7% were 19–25, 57.9% were 26–34, and 14.8% were ≥35 years of age. The hospitalization rates of pregnant women with HCV increased overall between 2010 and 2020, with the highest velocity in the 26–34 age group. The 26–34 age group had the highest HCV burden, with an age-standardized hospitalization rate of 660 per 100,000 in 2020. The rates of mortality and cirrhosis were significantly higher in the HCV cohort and increased further with age (*p* < 0.05). Among the HCV pregnant cohort, 151,017 (77.1%) delivered during hospitalization. Preterm births and stillbirths were significantly higher in the HCV pregnant cohort compared to the controls across multiple age groups (*p* < 0.05). Minority race/ethnicity was associated with increased mortality, cirrhosis, preterm birth, and stillbirth (*p* < 0.001). HIV co-infection, hepatitis B co-infection, and diabetes increased the odds of cirrhosis (*p* < 0.001). Conclusions: Hospitalizations of pregnant women with HCV are escalating, and these women are at increased risk of mortality, cirrhosis, preterm birth, and stillbirth with modifying factors, exacerbating risks further.

## 1. Introduction

Hepatitis C, which is caused by the hepatitis C virus (HCV), is a major contributor to chronic liver diseases worldwide [1]. In the United States, there are an estimated 3.5 million people currently infected with HCV [2], surpassing hepatitis B as the dominant viral hepatitis disease in this country, with a four times greater prevalence [3]. Globally, hepatitis B and hepatitis C account for approximately 96% of viral-hepatitis-related mortality and 91% of viral-hepatitis-related disability-adjusted life-years [4].

Within the pregnant population, it is estimated that 1–2.5% of pregnant women in the United States are infected with HCV [5]. Hepatitis C in pregnancy has been associated with several negative outcomes for the fetus/neonate, including low birth weight, small for gestational age, requiring assisted ventilation, and requiring admission to a neonatal intensive care unit [6]. However, for mothers with hepatitis C, the state of pregnancy has been associated with a degree of improvement in their disease, reflecting decreased alanine aminotransferase levels and overall reduced hepatic injury despite concurrently increased HCV RNA levels [7,8]. Nevertheless, pre-existing cirrhosis can still significantly negatively impact pregnancy; it is associated with worse outcomes for the mother, including developing intrahepatic cholestasis of pregnancy and puerperal infections [9].

While injection drug use remains the largest contributor to HCV transmission, another major route is vertical transmission, from the infected mother to the neonate/fetus during birth [10]. The vertical transmission rate is estimated to be 5.8% [5]. Infection of the neonate/fetus with HCV confers significant morbidity, such as liver fibrosis, cirrhosis, liver failure, glomerulonephritis, developmental delays, and cognitive deficits [11]. Additionally, because HCV infection in neonates/fetuses may be asymptomatic for years, liver damage may present without warning, or these individuals may unknowingly transmit HCV to others in their adolescence or adulthood. Chronic hepatitis C acquired in childhood is also associated with a 26-fold increased risk of liver-related death [12].

Understanding the burden of hepatitis C in the pregnant population is important. However, as reproductive technology continues to advance and women continue to delay pregnancy to older ages, there is also a need to understand the maternal and fetal outcomes in pregnant women with hepatitis C over a range of ages. Thus, we aim to describe the age-stratified clinical outcomes and trends for inpatient pregnant women with HCV and their fetuses/neonates in the United States.

## 2. Materials and Methods

### 2.1. Study Population

After receiving approval from the institutional review board of the Johns Hopkins University School of Medicine, we utilized data from the National Inpatient Sample, a database on inpatient stays and hospital discharges in the United States from the Healthcare Cost and Utilization Project (HCUP). The hospitalization data are de-identified, and individuals with multiple hospitalizations cannot be tracked. Data from 1 January 2010 to 31 December 2020 (the most recent year for which data were available) were extracted and analyzed.

To obtain our primary study cohort, we included all hospitalizations with a diagnosis of pregnancy and a concurrent diagnosis of hepatitis C from 1 January 2010 to 31 December 2020. We identified hospitalizations with pregnancy and hepatitis C using the International Classification of Diseases, Ninth Revision (ICD-9) and the International Classification of Diseases, Tenth Revision (ICD-10) diagnosis codes. Hepatitis C was defined according to the ICD-9 diagnostic codes 070.41 (acute hepatitis C with hepatic coma), 070.44 (chronic hepatitis C with hepatic coma), 070.51 (acute hepatitis C without mention of hepatic coma), 070.54 (chronic hepatitis C without mention of hepatic coma), 070.70 (unspecified viral hepatitis C without hepatic coma), and 070.71 (unspecified viral hepatitis C with hepatic coma) and the ICD-10 diagnostic codes B17.10 (acute hepatitis C without hepatic coma), B17.11 (acute hepatitis C with hepatic coma), B18.2 (chronic viral hepatitis C), B19.20 (unspecified viral hepatitis C without hepatic coma), and B19.21 (unspecified viral hepatitis C with hepatic coma). There were no exclusions based on age, race/ethnicity, or geography within the United States. The study cohort was stratified by age groups of 0–18, 19–25, 26–34, and 35+ years of age.

We extracted demographic and geographic data including age, sex, race/ethnicity, region of hospital, and type of hospital (rural, urban non-teaching, or urban teaching). The following clinical data were obtained: length of hospital stay, all-cause mortality, cirrhosis, hepatitis B virus (HBV) co-infection, HIV co-infection, history of injection drug use, obesity, diabetes mellitus (including type 1 and 2, but excluding gestational diabetes), delivery of infant during hospitalization, preterm birth (<37 weeks gestation), post-term birth (>41 weeks gestation), live birth, and stillbirth. We classified hospitalizations with a diagnosis of dependence on drugs commonly injected intravenously, including opioids, sedatives, amphetamines, hallucinogens, or combinations of these, as having a history of injection drug use.

### 2.2. Statistical Analysis

Hospitalization rates were analyzed for their trends in the 2010 to 2020 period using Poisson regression and reported as incidence rate ratios (IRRs) per year. The hospitalization rates involving our age groups were age-standardized, using the total inpatient pregnancies of each respective age group as the denominators. The hospitalization rate calculations incorporated the discharge weights provided by HCUP to provide the true number of hospitalizations, compensating for the 20% sampling of the database. Changes in the discharge weights prior to 2012 due to database redesign were accounted for in the hospitalization rates. The case fatality rates were calculated as the percentage of deaths that resulted from all hospitalizations for a given disease. Quantitative data were tested for normality using the Kolmogorov–Smirnov test. The non-normal data were summarized using medians and interquartile ranges (IQRs) and compared using the Mann–Whitney U test. Normally distributed data were analyzed using Student’s *t*-test. Frequencies were compared using the chi-squared test. The risk factors for same-visit mortality and developing cirrhosis among the inpatient HCV pregnant women were analyzed using logistic regression in both univariable (crude) and multivariable (adjusted) models. Among the inpatient HCV pregnant women who delivered during their hospitalization, the risk factors for preterm birth and stillbirth were also analyzed using logistic regression in both univariable and multivariable models. The results from the multivariable model were reported. The results of the logistic regression were reported as odds ratios (ORs) with 95% confidence intervals (CI). Odds ratios from continuous data such as age represent the increase in odds for each unit increase (i.e., 1-year increase in age).

All the results reported were weighted, using the discharge weights provided by HCUP to present the true number of hospitalizations in the United States. Results for a category that contained 10 or fewer hospitalizations but greater than zero hospitalizations were displayed as ≤10 due to the data use privacy policy of HCUP. The *p*-values were calculated from the weighted data using actual numbers regardless of whether ≤10 hospitalizations were reported. Missing data were assumed to be missing at random and excluded from the statistical analyses. All reported *p*-values were two-tailed *p*-values. Statistical significance was defined as *p* < 0.05. The Benjamin–Hochberg procedure was applied to control for multiple comparisons with a selected false discovery rate of 5%. Any comparison that was not found to be significant after applying the Benjamin–Hochberg procedure despite having *p* < 0.05 was reported as being non-significant. The statistical calculations were performed using the R program (R Core Team (2020). R: A language and environment for statistical computing. R Foundation for Statistical Computing, Vienna, Austria. URL https://www.R-project.org/ (accessed on 15 January 2024)).

## 3. Results

Out of a total of 44,636,244 pregnant women who were hospitalized between 2010 and 2020, we identified 195,852 with HCV and 44,440,393 without HCV. In the cohort with HCV, the median age was 29 (IQR: 25–32) years, compared to 28 (IQR: 24–33) years in the cohort without HCV (*p* < 0.001). The median length of hospitalization stay was 3 (IQR: 2–3) days and 2 (IQR: 2–3) days in the cohorts with HCV and without HCV, respectively (*p* < 0.001). The ethnicity/race distribution of the pregnant women with HCV was 150,801 (77.0%) non-Hispanic White, 11,131 (5.7%) non-Hispanic Black, 14,383 (7.3%) Hispanic, 2207 (1.1%) Asian or Pacific Islander, 3126 (1.6%) Native American, and 14,203 (7.3%) other/unknown. Compared to the cohort of pregnant women without HCV, the cohort with HCV had a significantly larger proportion of non-Hispanic White patients (*p* < 0.001). Most of the hospitalizations from the HCV cohort took place in the Southern region (44.9%) of the United States, and most occurred in urban teaching hospitals (68.3%). Mortality was significantly higher in the HCV cohort compared to the non-HCV cohort, with 209 (0.107%) versus 8211 (0.018%) dying during hospitalization, respectively (*p* < 0.001) (Table 1).

The age group distribution trend followed a similar pattern for the HCV and non-HCV cohorts. Among the cohort of pregnant women with HCV, 0.7% were ≤18, 26.7% were 19–25, 57.9% were 26–34, and 14.8% were ≥35 years of age. In contrast, the age group distribution of the non-HCV pregnant cohort was significantly different; a total of 3.4% were ≤18, 29.3% were 19–25, 50.7% were 26–34, and 16.5% were ≥35 years of age (*p* < 0.001).

**Figure 1:**
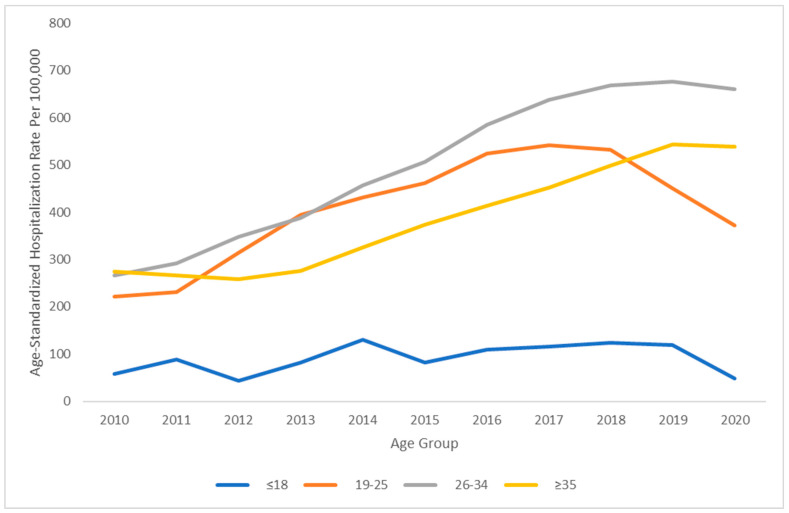
Nationwide hospitalization rates of pregnant women with hepatitis C from 2010 to 2020, stratified by age group.

The hospitalization rates of pregnant women with HCV increased overall between 2010 and 2020, with an annual incidence rate ratio of 1.09 (95% CI: 1.09–1.09; *p* < 0.001), which is approximately equivalent to a 9% increase in the hospitalization rate each year on average. When stratified by age group, the Poisson regression analysis revealed distinct hospitalization trends for the age groups. The hospitalizations rates of pregnant women with HCV increased in the ≤18 age group (IRR: 1.04; 95% CI: 1.01–1.08; *p* = 0.015), the 19–25 age group (IRR: 1.06; 95% CI: 1.06–1.07; *p* < 0.001), the 26–34 age group (IRR: 1.10; 95% CI: 1.09–1.10; *p* < 0.001), and the ≥35 age group (IRR: 1.09; 95% CI: 1.08–1.10; *p* < 0.001). The 26–34 age group had the highest HCV hospitalization burden, with an age-standardized hospitalization rate of 660 per 100,000 inpatient pregnant women in 2020 (Figure 1).

**Figure 2:**
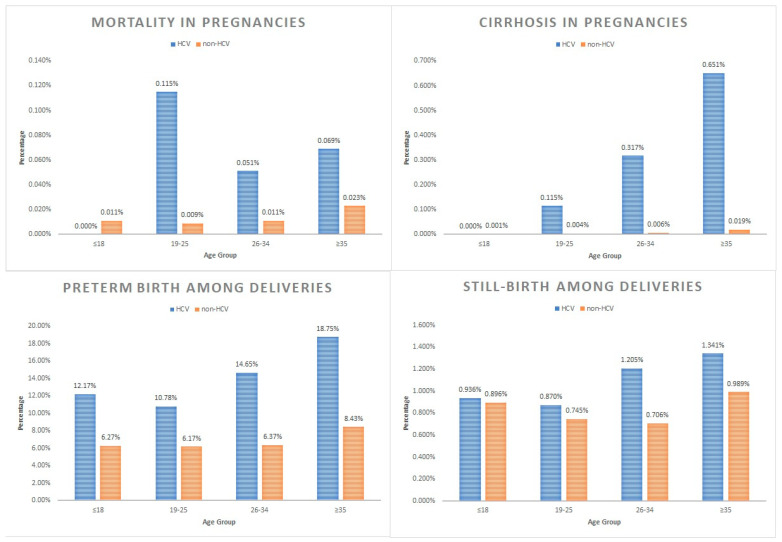
Comparative frequencies of mortality and liver cirrhosis among inpatient pregnant women and preterm birth and stillbirth among inpatient pregnant women who delivered, stratified by hepatitis C virus (HCV) infection status, 2010–2020.

Overall, the maternal mortality rate of the HCV pregnant cohort was 0.107% compared to 0.018% in the non-HCV pregnant cohort (*p* < 0.001). When stratified by age group, the maternal mortality rates in the HCV pregnant cohort were similar to the non-HCV pregnant cohort for the ≤18 age group (0.000% vs. 0.011%; *p* = 0.866). However, the maternal mortality rates were significantly higher in the HCV pregnant cohort for the 19–25 age group (0.115% vs. 0.009%; *p* < 0.001), the 26–34 age group (0.051% vs. 0.011%; *p* < 0.001), and the ≥35 age group (0.069% vs. 0.023%; *p* = 0.020) compared to the respective non-HCV pregnant sub-cohorts. The rates of cirrhosis development were similar between the HCV and non-HCV pregnant cohorts for the ≤18 age group (0.000% vs. 0.001%; *p* = 0.953). However, the rates of cirrhotic sequalae were significantly higher in the HCV pregnant cohort for the 19–25 age group (0.115% vs. 0.004%: *p* < 0.001), the 26–34 age group (0.317% vs. 0.006%; *p* < 0.001), and the ≥35 age group (0.651% vs. 0.019%; *p* < 0.001), with a clear upward trend as age increased (Figure 2).

We further explored the cirrhosis subgroup. Among the HCV pregnant cohort, there were a total of 817 (0.42%) pregnancies involving cirrhosis. Among this subgroup, 35 (4.284%) died during their hospitalization. This mortality rate was also slightly higher compared to the subgroup of pregnancy involving cirrhosis without HCV, who had a mortality rate of 3.571%, although this difference was not statistically significant (*p* = 0.669). There were only 20 (2.4%) cases of pre-eclampsia in the pregnant HCV cirrhosis group, none of whom died. There were no cases of eclampsia in the pregnant HCV cirrhosis group.

Among the HCV pregnant cohort, 151,017 (77.1%) delivered during their hospitalization stay. The rates of preterm birth in the HCV cohort were significantly higher than in the non-HCV cohort for all age groups: the ≤18 age group (12.17% vs. 6.27%; *p* < 0.001), the 19–25 age group (10.78% vs. 6.17%; *p* < 0.001), the 26–34 age group (14.65% vs. 6.37%; *p* < 0.001), and the ≥35 age group (18.75% vs. 8.43%; *p* < 0.001). There was also an upward trend in the preterm birth rates in the HCV cohort as age increased. The rates of stillbirths in the HCV cohort were similar to those of the non-HCV cohort for the ≤18 age group (0.936% vs. 0.896%; *p* = 0.949) and the 19–25 age group (0.870% vs. 0.745%; *p* = 0.187). However, the rates of stillbirth were significantly higher in the HCV cohort for the 26–34 age group (1.205% vs. 0.706%; *p* < 0.001) and the ≥35 age group (1.341% vs. 0.989%; *p* = 0.022) (Figure 2).

Using a multivariable logistic regression model, we explored the risk factors that increased the odds of maternal mortality and cirrhosis within the HCV pregnant cohort. Increased maternal age (OR: 1.04; 95% CI: 1.04–1.04; *p* < 0.001) and the presence of HBV co-infection (OR: 2.57; 95% CI: 1.75–3.77; *p* < 0.001) were associated with increased odds of maternal mortality. However, non-Hispanic White race/ethnicity (OR: 0.86; 95% CI: 0.81–0.91; *p* < 0.001) and obesity (OR: 0.70; 95% CI: 0.64–0.77; *p* < 0.001) were associated with decreased odds of maternal mortality. The presence of HIV or diabetes was not significantly associated with maternal mortality (Table 2). Additionally, increased age (OR: 1.01; 95% CI: 1.01–1.02; *p* < 0.001), HIV co-infection (OR: 2.22; 95% CI: 1.72–2.88; *p* < 0.001), HBV co-infection (OR: 13.41; 95% CI: 10.85–16.57; *p* < 0.001), and diabetes mellitus (OR: 1.83; 95% CI: 1.72–1.95; *p* < 0.001) were significantly associated with increased odds of developing cirrhosis. In contrast, non-Hispanic White race/ethnicity (OR: 0.91; 95% CI: 0.86–0.97; *p* = 0.003) was associated with decreased odds of developing cirrhosis. The presence of obesity was not associated with developing cirrhosis (Table 3).

Additionally, we explored the risk factors that increased the odds of preterm birth and stillbirth within the HCV pregnant cohort that delivered during hospitalization. Obesity (OR: 1.65; 95% CI: 1.46–1.88; *p* < 0.001) was significantly associated with increased odds of preterm birth. However, non-Hispanic White race/ethnicity (OR: 0.74; 95% CI: 0.69–0.78; *p* < 0.001), HIV co-infection (OR: 0.24; 95% CI: 0.09–0.65; *p* = 0.005), and diabetes mellitus (OR: 0.30; 95% CI: 0.25–0.37; *p* < 0.001) were associated with decreased odds of preterm birth. The presence of HBV co-infection was not associated with preterm birth (Table 4). Furthermore, non-Hispanic White race/ethnicity (OR: 0.52; 95% CI: 0.39–0.69; *p* < 0.001), and diabetes mellitus (OR: 0.23; 95% CI: 0.11–0.49; *p* < 0.001) were significantly associated with decreased odds of stillbirth. The presence of obesity was not associated with stillbirth (Table 5).

Of note, out of the total 195,851 pregnancies involving HCV, there were 2223 (1.2%) pregnancies with HBV co-infection compared to 66,886 (0.2%) in the non-HCV pregnant cohort (*p* < 0.001). Additionally, there were 2197 (1.1%) pregnancies with HIV co-infection in the HCV cohort compared to 49,578 (0.1%) in the non-HCV cohort (*p* < 0.001). Furthermore, we found that in the HCV pregnant cohort, 78,786 (40.2%) pregnancies involved injection drug use compared to 350,362 (0.8%) in the non-HCV pregnant cohort (*p* < 0.001). Upon further investigation, we found no associations between injection drug use and mortality within the HCV pregnant cohort; a total of 35.9% of patients that died and 40.2% of patients that survived within the HCV pregnant cohort had associated injection drug use (*p* = 0.555). However, among the HCV pregnant cohort who delivered, 50.3% of preterm births versus 32.1% of non-preterm births had associated injection drug use (*p* < 0.001).

## 4. Discussion

This present study is one of the largest of its kind and provides important data on the health burden of hepatitis C in pregnant women and their fetuses/neonates over a range of ages. Overall, we found that the hospitalization rates of pregnant women with HCV increased over time from 2010 to 2020. This concerning trend is consistent with prior studies as well [13]. Additionally, we found that hospitalization rates of pregnant women with HCV have also increased across all age groups and increased most rapidly in the 26–34 age group. Furthermore, the 26–34 age group also had the highest burden of pregnant women with HCV, having the highest hospitalization rate among the four age groups investigated.

Importantly, we found that the overall mortality was higher in pregnant women with HCV, and this increase in mortality became significant in the age groups above 18 years of age. Additionally, we found that the rates of stillbirths in pregnant women with HCV became significantly higher than in pregnant women without HCV after 25 years of age (1.2–1.3% vs. 0.7–1.0%). Prior studies exploring maternal mortality and stillbirths in the context of HCV have been largely limited or inconclusive, potentially due to insufficient sample sizes, as maternal and neonatal mortality in the United States, in general, is rare. A recent systematic review and meta-analysis by Shen et al. similarly found the rates of maternal mortality and neonatal mortality to be higher in pregnant women with HCV compared to those without HCV [14], although they did not explore age-stratified mortality, as in this present study. In counseling pregnant women about HCV infection during pregnancy, physicians should explain that age does concur a gradual but statistically significant clinical increased risk of maternal death, 1.04-fold for every year increase in maternal age; furthermore, co-infection with HBV does further increase this risk 2.6 fold. As the average age of first-time pregnancies is increasing over time in the United States, and advances in reproductive technology and medicine are increasing the upper age limit of viable pregnancies, the contribution of age to mortality in pregnant women with HCV could become increasingly more clinically significant over time.

HCV infection in pregnant women is known to be associated with an increased risk of preterm birth [15]. Similarly, we found that pregnant women with HCV had higher frequencies of preterm births (10.78–18.75%), but we also found that this was true across all age groups, and the rates of preterm births increased as maternal age increased. In the general population, teen pregnancies are associated with an increased risk of preterm birth (8.5–14%) compared to that of older pregnant women (10.2%) [16,17,18]. In our study, the rate of preterm birth was the highest among the older women, ranking as follows: the ≥35 age group (18.75%); the 26–34 age group (14.65%); the 19–25 age group (10.78%); and the ≤18 age group (12.17%). This may reflect a longer term of HCV infection with increasing maternal health complications that contributed to the risk of spontaneous or medical indicated preterm birth. Additionally, we found injection drug use to be more common in pregnant women with HCV who had preterm birth compared to those without preterm birth, which may have contributed to the preterm birth findings; however, it is unclear whether injection drug use itself directly causes preterm births or it is simply a proxy for worse socioeconomic status or healthcare compliance.

In pregnant women with HCV, we found after the age of 18, cirrhosis became significantly more common compared to in the non-HCV controls. Importantly, we found a clear upward trend in the rates of cirrhosis as age increased in pregnant women with HCV, from 0.115% at age 19–25 to 0.651% at age ≥35. Cirrhosis is an uncommon finding in young patients, even among those with HCV infection [19]. To put our findings into perspective, a cirrhosis rate of 0.651% is approximately 1 per 150 pregnant women with HCV at an age ≥35 years. This is a six-fold increase compared to the 19–25 age group with HCV and a 34-fold increase compared to non-HCV pregnant women of the same age group. Furthermore, pregnant women with HCV and cirrhosis had notably much higher rates of mortality (4.284%) compared to the general HCV pregnant cohort (0.107%). However, potentially due to the limited sample size of this sub-cohort, we did not find a significant difference in the mortality rates of the group of pregnant women with cirrhosis and HCV compared to the group of pregnant women with cirrhosis but without HCV, who had a mortality rate of 3.571%. Nevertheless, this highlights the clinical importance of cirrhosis to pregnancy management and outcomes. Hepatitis-C-associated cirrhosis in pregnant women across a range of ages has not been well studied, but cirrhosis, in general, is known to be also associated with an increased risk of cesarean delivery, low birth weight, and preterm delivery [20].

For all the outcomes we explored (i.e., mortality, cirrhosis, preterm birth, and stillbirth), we found that pregnant women who belonged to a minority race/ethnicity had worse outcomes. This is largely consistent with general studies exploring race/ethnicity in pregnancy [21,22,23]. However, the role of race/ethnicity in the development of cirrhosis in pregnant women has not been previously explored. We found that patients with concomitant HCV and HIV or diabetes were less likely to experience preterm delivery. These results are somewhat surprising; however, recognizing those diagnosed with diabetes are vigorously treated and monitored during their pregnancies to a greater extent than those without diabetes, this reduction in preterm delivery could reflect increased surveillance. This close surveillance, which can involve weekly to twice-weekly fetal testing (i.e., nonstress tests or biophysical profiles), may also explain the decreased risk of stillbirth in pregnant women with HCV and diabetes. This suggests that pregnant women with HCV, in general, potentially benefit from more frequent prenatal clinic visits and closer surveillance. In some prior studies of pregnant women with HIV, the investigators have reported that HIV was associated with an increased risk of preterm birth [24,25,26]. The reduction in preterm delivery in those with HCV and HIV warrants further study; HIV infection has, in some, but not all, series, demonstrated an increased risk of preterm delivery despite antiretroviral therapy [27]. The complex immunomodulation of pregnancy and the interaction of HIV and HCV could potentially lead to a decreased risk of preterm birth.

Furthermore, we also found that in pregnant women with HCV infection, HBV co-infection was very strongly associated with cirrhosis development, increasing its risk approximately 13-fold. Liver fibrosis and the development of cirrhosis are known to be accelerated in non-pregnant patients who have HCV and HBV co-infection [28]. Similarly, HIV co-infection is known to accelerate the progression of liver fibrosis in non-pregnant patients with HCV infection [29,30]. This is consistent with our findings of increased odds of cirrhosis in pregnant women with HCV with HIV co-infection.

Of note, in this study, we found an unusually low percentage of non-Hispanic Black pregnant women in our HCV cohort compared to our non-HCV cohort, 5.7% versus 14.8%, respectively. In a large multicenter study of over 8000 adult patients with HCV by Gordon et al., the authors reported that Black patients comprised approximately 23.8% of their cohort. This disparity may indicate a significant underdiagnosis of HCV in the pregnant non-Hispanic Black population. Alternatively, this may also indicate that pregnant non-Hispanic Black patients with HCV could be less likely to choose to deliver in a hospital setting, or they may be less likely to have access to a hospital with obstetricians. Nevertheless, considering the increasing rates of HCV in pregnant women, a stronger public health response to find and treat HCV is needed in general for young women of reproductive age and particularly in communities of non-Hispanic Black populations.

There were a few limitations in this study. Firstly, the National Inpatient Sample is a retrospective database and thus has the inherent limitations and biases of retrospective studies. Additionally, the database does not have standardized diagnostic criteria. We have also relied on the professional judgment of clinicians in recording the most appropriate diagnostic codes. Furthermore, there was a possibility of miscoding. For this study, we assumed that any miscoded diagnoses and differing coding behaviors amongst clinicians would be randomly distributed and would not result in statistical significance. Another limitation of database analysis is that it cannot account for the varied genotypes and subtypes of HCV. HCV has 11 major genotypes with 15 different subtypes, and their prevalence in different regions of the world varies [31]. Their biological effects, such as the severity of damage caused by each major genotype, do differ significantly [32,33]. Socioeconomic status and access to healthcare, including prenatal care, are factors that can influence maternal and fetal outcomes in addition to HCV infection itself. We were unable to adjust for these factors in our study.

## 5. Conclusions

Overall, the hospitalization rates of pregnant women with HCV have increased over time and across all age groups, most rapidly in the 26–34 age group. Mortality, cirrhosis, preterm births, and stillbirths were more common in general in pregnant women with HCV than in non-HCV pregnant women, with age group being a modifying factor. The 35+ age group had the highest health burden compared to the other age groups, being associated with high rates of mortality, cirrhosis, preterm births, and stillbirths. Minority race/ethnicity was associated with worse outcomes in terms of mortality, cirrhosis, preterm births, and stillbirths. HIV co-infection, HBV co-infection, and diabetes increased the odds of cirrhosis in pregnant women with HCV. Those managing pregnant patients with HCV should remain watchful of these potential medical and obstetric hazards. Further studies are needed to better elucidate the causal or proxy relationships of the significant demographic and clinical factors we have described.

## Figures and Tables

**Table 1:** Demographic and geographic characteristics of nationwide pregnancy hospitalizations involving hepatitis C and without involvement of hepatitis C. National Inpatient Sample, Healthcare Cost and Utilization Project (HCUP), 2010–2020.

Characteristics	Hepatitis C	Non-Hepatitis C	*p*-Value
Number of Hospitalizations, N	195,851	44,440,393	
Age, Years, Median (IQR)	29 (25–32)	28 (24–33)	**0.021**
Length of Stay, Days, Median (IQR)	3 (2–3)	2 (2–3)	**<0.001**
Mortality, N (%)	209 (0.1)	8211 (<0.1)	**<0.001**
Race/Ethnicity			**<0.001**
Non-Hispanic White, N (%)	150,801 (77.0)	21,676,105 (48.8)	
Non-Hispanic Black, N (%)	11,131 (5.7)	6,588,504 (14.8)	
Hispanic, N (%)	14,383 (7.3)	8,892,075 (20.0)	
Asian or Pacific Islander, N (%)	2207 (1.1)	2,354,304 (5.3)	
Native American, N (%)	3126 (1.6)	327,003 (0.7)	
Other/Unknown, N (%)	14,203 (7.3)	4,602,402 (10.4)	
Region of Hospital			**<0.001**
Northeast, N (%)	39,777 (20.3)	7,132,807 (16.1)	
Midwest, N (%)	40,467 (20.7)	9,305,085 (20.9)	
South, N (%)	87,959 (44.9)	17,272,945 (38.9)	
West, N (%)	27,647 (14.1)	10,729,556 (24.1)	
Type of Hospital			**<0.001**
Rural, N (%)	26,802 (13.7)	4,307,036 (9.7)	
Urban Non-Teaching, N (%)	34,438 (17.6)	12,549,502 (28.2)	
Urban Teaching, N (%)	133,737 (68.3)	27,444,930 (61.8)	
Unknown, N (%)	874 (0.4)	138,925 (0.3)	

Bolded *p*-values are statistically significant.

**Table 2:** Logistic regression of demographic and clinical factors for association with mortality within a cohort of pregnant women with hepatitis C virus infection.

Risk Factors		Crude Odds Ratio (95% CI)	Crude *p*-Value	Adjusted Odds Ratio (95% CI)	Adjusted *p*-Value
Demographics					
Age	High (vs. Low)	1.04 (1.04–1.04)	**<0.001**	1.04 (1.04–1.04)	**<0.001**
Race/Ethnicity	White (vs. Other)	1.30 (1.23–1.37)	**<0.001**	0.86 (0.81–0.91)	**<0.001**
Co-Infection					
HIV	Yes/No	0.78 (0.54–1.13)	0.190	0.92 (0.62–1.36)	0.666
HBV	Yes/No	2.43 (1.68–3.50)	**<0.001**	2.57 (1.75–3.77)	**<0.001**
Co-Morbidity					
Diabetes	Yes/No	1.58 (1.50–1.67)	**<0.001**	1.03 (0.97–1.09)	0.326
Obesity	Yes/No	0.73 (0.67–0.80)	**<0.001**	0.70 (0.64–0.77)	**<0.001**

CI = Confidence Interval; HBV = Hepatitis B Virus; HIV = Human Immunodeficiency Virus. The multivariable (adjusted) logistic regression model included all the variables listed in the table for adjustment. Bolded *p*-values are statistically significant.

**Table 3:** Logistic regression of demographic and clinical factors for association with cirrhosis within a cohort of pregnant women with hepatitis C virus infection.

Risk Factors		Crude Odds Ratio (95% CI)	Crude *p*-Value	Adjusted Odds Ratio (95% CI)	Adjusted *p*-Value
Demographics					
Age	High (vs. Low)	1.02 (1.02–1.02)	**<0.001**	1.01 (1.01–1.02)	**<0.001**
Race/Ethnicity	White (vs. Other)	1.02 (0.96–1.08)	0.475	0.91 (0.86–0.97)	**0.003**
Co-Infection					
HIV	Yes/No	2.85 (2.27–3.57)	**<0.001**	2.22 (1.72–2.88)	**<0.001**
HBV	Yes/No	15.70 (12.92–19.09)	**<0.001**	13.41 (10.85–16.57)	**<0.001**
Co-Morbidity					
Diabetes	Yes/No	2.36 (2.23–2.49)	**<0.001**	1.83 (1.72–1.95)	**<0.001**
Obesity	Yes/No	1.20 (1.10–1.30)	**<0.001**	0.98 (0.89–1.07)	0.602

CI = Confidence Interval; HBV = Hepatitis B Virus; HIV = Human Immunodeficiency Virus. The multivariable (adjusted) logistic regression model included all the variables listed in the table for adjustment. Bolded *p*-values are statistically significant.

**Table 4:** Logistic regression of demographic and clinical factors for association with preterm birth within a cohort of pregnant women with hepatitis C virus infection who delivered during hospitalization.

Risk Factors		Crude Odds Ratio (95% CI)	Crude *p*-Value	Adjusted Odds Ratio (95% CI)	Adjusted *p*-Value
Demographics					
Age	High (vs. Low)	0.96 (0.96–0.96)	**<0.001**	0.96 (0.96–0.96)	**<0.001**
Race/Ethnicity	White (vs. Other)	0.49 (0.46–0.52)	**<0.001**	0.74 (0.69–0.78)	**<0.001**
Co-Infection					
HIV	Yes/No	0.17 (0.06–0.44)	**<0.001**	0.24 (0.09–0.65)	**0.005**
HBV	Yes/No	0.68 (0.30–1.52)	0.346	1.49 (0.67–3.33)	0.328
Co-Morbidity					
Diabetes	Yes/No	0.10 (0.09–0.12)	**<0.001**	0.30 (0.25–0.37)	**<0.001**
Obesity	Yes/No	0.71 (0.63–0.80)	**<0.001**	1.65 (1.46–1.88)	**<0.001**

CI = Confidence Interval; HBV = Hepatitis B Virus; HIV = Human Immunodeficiency Virus. The multivariable (adjusted) logistic regression model included all the variables listed in the table for adjustment. Bolded *p*-values are statistically significant.

**Table 5:** Logistic regression of demographic and clinical factors for association with stillbirth within a cohort of pregnant women with hepatitis C virus infection who delivered during hospitalization.

Risk Factors		Crude Odds Ratio (95% CI)	Crude *p*-Value	Adjusted Odds Ratio (95% CI)	Adjusted *p*-Value
Demographics					
Age	High (vs. Low)	0.97 (0.97–0.98)	**<0.001**	0.98 (0.98–0.98)	**<0.001**
Race/Ethnicity	White (vs. Other)	0.41 (0.31–0.53)	**<0.001**	0.52 (0.39–0.69)	**<0.001**
Co-Infection					
HIV	Yes/No	1.61 (0.40–6.47)	0.504	1.65 (0.40–6.75)	0.484
HBV	Yes/No	N/A	N/A	N/A	N/A
Co-Morbidity					
Diabetes	Yes/No	0.13 (0.07–0.27)	**<0.001**	0.23 (0.11–0.49)	**<0.001**
Obesity	Yes/No	0.73 (0.45–1.20)	0.215	1.21 (0.72–2.04)	0.467

CI = Confidence Interval; HBV = Hepatitis B Virus; HIV = Human Immunodeficiency Viru. The multivariable (adjusted) logistic regression model included all variables listed in the table for adjustment. Bolded *p*-values are statistically significant.

## Data Availability

The data presented in this study are available on request from the corresponding author. The data are not publicly available due to patient confidentiality.

## References

[1] Roudot-Thoraval F. (2021). Epidemiology of hepatitis C virus infection. Clin. Res. Hepatol. Gastroenterol..

[2] Edlin B.R., Eckhardt B.J., Shu M.A., Holmberg S.D., Swan T. (2015). Toward a more accurate estimate of the prevalence of hepatitis C in the United States. J. Hepatol..

[3] Roberts H., Kruszon-Moran D., Ly K.N., Hughes E., Iqbal K., Jiles R.B., Holmberg S.D. (2016). Prevalence of chronic hepatitis B virus (HBV) infection in U.S. households: National Health and Nutrition Examination Survey (NHANES), 1988–2012. J. Hepatol..

[4] Stanaway J.D., Flaxman A.D., Naghavi M., Fitzmaurice C., Vos T., Abubakar I., Abu-Raddad L.J., Assadi R., Bhala N., Cowie B. (2016). The global burden of viral hepatitis from 1990 to 2013: Findings from the Global Burden of Disease Study 2013. Lancet.

[5] Benova L., Mohamoud Y.A., Calvert C., Abu-Raddad L.J. (2014). Vertical Transmission of Hepatitis C Virus: Systematic Review and Meta-analysis. Clin. Infect. Dis..

[6] Pergam S.A., Wang C.C., Gardella C.M., Sandison T.G., Phipps W.T., Hawes S.E. (2008). Pregnancy Complications Associated with Hepatitis C: Data from a 2003–2005 Washington State Birth Cohort. Am. J. Obstet. Gynecol..

[7] Di Martino V., Lebray P., Myers R.P., Pannier E., Moussalli J., Thabut D., Buffet C., Poynard T., Paradis V., Charlotte F. (2004). Progression of liver fibrosis in women infected with hepatitis C: Long-term benefit of estrogen exposure. J. Hepatol..

[8] Gervais A., Bacq Y., Bernuau J., Martinot M., Auperin A., Boyer N., Kilani A., Erlinger S., Valla D., Marcellin P. (2000). Decrease in serum ALT and increase in serum HCV RNA during pregnancy in women with chronic hepatitis C. J. Hepatol..

[9] Flemming J.A., Mullin M., Lu J., Sarkar M.A., Djerboua M., Velez M.P., Brogly S., Terrault N.A. (2020). Outcomes of Pregnant Women With Cirrhosis and Their Infants in a Population-Based Study. Gastroenterology.

[10] Hagan L.M., Schinazi R.F. (2013). Best strategies for global HCV eradication. Liver Int..

[11] Mack C.L., Gonzalez-Peralta R.P., Gupta N., Leung D., Narkewicz M.R., Roberts E.A., Rosenthal P., Schwarz K.B. (2012). NASPGHAN Practice guidelines: Diagnosis and management of hepatitis c infection in infants, children, and adolescents. J. Pediatr. Gastroenterol. Nutr..

[12] Omland L.H., Krarup H., Jepsen P., Georgsen J., Harritshøj L.H., Riisom K., Jacobsen S.E.H., Schouenborg P., Christensen P.B., Sørensen H.T. (2010). Mortality in patients with chronic and cleared hepatitis C viral infection: A nationwide cohort study. J. Hepatol..

[13] Patrick S.W., Bauer A.M., Warren M.D., Jones T.F., Wester C. (2017). Hepatitis C Virus Infection Among Women Giving Birth—Tennessee and United States, 2009–2014. MMWR. Morb. Mortal. Wkly. Rep..

[14] Shen G.-F., Ge C.-H., Shen W., Liu Y.-H., Huang X.-Y. (2023). Association between hepatitis C infection during pregnancy with maternal and neonatal outcomes: A systematic review and meta-analysis. Eur. Rev. Med. Pharmacol. Sci..

[15] Seto M.T.-Y., Cheung K.W., Hung I.F. (2020). Management of viral hepatitis A, C, D and E in pregnancy. Best Pract. Res. Clin. Obstet. Gynaecol..

[16] Khashan A.S., Baker P.N., Kenny L.C. (2010). Preterm birth and reduced birthweight in first and second teenage pregnancies: A register-based cohort study. BMC Pregnancy Childbirth.

[17] Baker A.M., Haeri S. (2014). Estimating risk factors for spontaneous preterm delivery in teen pregnancies. Arch. Gynecol. Obstet..

[18] (2021). Prediction and Prevention of Spontaneous Preterm Birth: ACOG Practice Bulletin, Number 234. Obstet. Gynecol..

[19] Freeman A.J., Dore G.J., Law M.G., Thorpe M., Von Overbeck J., Lloyd A.R., Marinos G., Kaldor J.M. (2001). Estimating progression to cirrhosis in chronic hepatitis C virus infection. J. Hepatol..

[20] Hagström H., Höijer J., Marschall H., Williamson C., Heneghan M.A., Westbrook R.H., Ludvigsson J.F., Stephansson O. (2018). Outcomes of Pregnancy in Mothers With Cirrhosis: A National Population-Based Cohort Study of 1.3 Million Pregnancies. Hepatol. Commun..

[21] Venkatesh K.K., Lynch C.D., Powe C.E., Costantine M.M., Thung S.F., Gabbe S.G., Grobman W.A., Landon M.B. (2022). Risk of Adverse Pregnancy Outcomes Among Pregnant Individuals With Gestational Diabetes by Race and Ethnicity in the United States, 2014–2020. JAMA.

[22] Dongarwar D., Ajewole V., Spooner K.K., Tran V., Adebusuyi T., Onyenaka C., Bakare O., Emeh C., Baines K., Boua D. (2023). Racial and Ethnic Disparities in Stillbirth among Pregnant Women with Obesity. Am. J. Perinatol..

[23] MacDorman M.F., Thoma M., Declcerq E., Howell E.A. (2021). Racial and Ethnic Disparities in Maternal Mortality in the United States Using Enhanced Vital Records, 2016–2017. Am. J. Public Health.

[24] Wedi C.O.O., Kirtley S., Hopewell S., Corrigan R., Kennedy S.H., Hemelaar J. (2016). Perinatal outcomes associated with maternal HIV infection: A systematic review and meta-analysis. Lancet HIV.

[25] Ravindran J., Richardson B.A., Kinuthia J., Unger J.A., Drake A.L., Osborn L., Matemo D., Patterson J., McClelland R.S., John-Stewart G. (2021). Chlamydia, Gonorrhea, and Incident HIV Infection During Pregnancy Predict Preterm Birth Despite Treatment. J. Infect. Dis..

[26] Malaba T.R., Phillips T., Le Roux S., Brittain K., Zerbe A., Petro G., Ronan A., McIntyre J.A., Abrams E.J., Myer L. (2017). Antiretroviral therapy use during pregnancy and adverse birth outcomes in South African women. Int. J. Epidemiol..

[27] Mugo C., Nduati R., Osoro E., Nyawanda B.O., Mirieri H., Hunsperger E., Verani J.R., Jin H., Mwaengo D., Maugo B. (2022). Comparable Pregnancy Outcomes for HIV-Uninfected and HIV-Infected Women on Antiretroviral Treatment in Kenya. J. Infect. Dis..

[28] Pol S., Haour G., Fontaine H., Dorival C., Petrov-Sanchez V., Bourliere M., Capeau J., Carrieri P., Larrey D., Larsen C. (2017). The negative impact of HBV/HCV coinfection on cirrhosis and its consequences. Aliment. Pharmacol. Ther..

[29] Pineda J.A., Romero-Gómez M., Díaz-García F., Girón-González J.A., Montero J.L., Torre-Cisneros J., Andrade R.J., González-Serrano M., Aguilar J., Aguilar-Guisado M. (2005). HIV coinfection shortens the survival of patients with hepatitis C virus-related decompensated cirrhosis. J. Hepatol..

[30] Graham C.S., Baden L.R., Yu E., Mrus J.M., Carnie J., Heeren T., Koziel M.J. (2001). Influence of Human Immunodeficiency Virus Infection on the Course of Hepatitis C Virus Infection: A Meta-Analysis. Clin. Infect. Dis..

[31] Keikha M., Eslami M., Yousefi B., Ali-Hassanzadeh M., Kamali A., Yousefi M., Karbalaei M. (2020). HCV genotypes and their determinative role in hepatitis C treatment. VirusDisease.

[32] Farci P., Purcell R.H. (2000). Clinical significance of hepatitis C virus genotypes and quasispecies. Semin. Liver Dis..

[33] Chilaka V.N., Konje J.C. (2021). Viral Hepatitis in pregnancy. Eur. J. Obstet. Gynecol. Reprod. Biol..

